# miR396-*OsGRF*s Module Balances Growth and Rice Blast Disease-Resistance

**DOI:** 10.3389/fpls.2018.01999

**Published:** 2019-01-14

**Authors:** Viswanathan Chandran, He Wang, Feng Gao, Xiao-Long Cao, Yun-Ping Chen, Guo-Bang Li, Yong Zhu, Xue-Mei Yang, Ling-Li Zhang, Zhi-Xue Zhao, Jing-Hao Zhao, Ying-Ge Wang, Shuangcheng Li, Jing Fan, Yan Li, Ji-Qun Zhao, Shao-Qing Li, Wen-Ming Wang

**Affiliations:** ^1^Rice Research Institute, Sichuan Agricultural University, Chengdu, China; ^2^State Key Laboratory of Hybrid Rice, Key Laboratory for Research and Utilization of Heterosis in Indica Rice of Ministry of Agriculture, College of Life Sciences, Wuhan University, Wuhan, China

**Keywords:** disease resistance, fitness cost, miR396, *Oryza sativa*, *OsGRF*, rice blast disease

## Abstract

Fitness cost is a common phenomenon in rice blast disease-resistance breeding. MiR396 is a highly conserved microRNA (miRNA) family targeting *Growth Regulating Factor* (*OsGRF*) genes. Mutation at the target site of miR396 in certain *OsGRF* gene or blocking miR396 expression leads to increased grain yield. Here we demonstrated that fitness cost can be trade-off in miR396-*OsGRF*s module via balancing growth and immunity against the blast fungus. The accumulation of miR396 isoforms was significantly increased in a susceptible accession, but fluctuated in a resistant accession upon infection of *Magnaporthe oryzae*. The transgenic lines over-expressing different miR396 isoforms were highly susceptible to *M. oryzae*. In contrast, overexpressing target mimicry of miR396 to block its function led to enhanced resistance to *M. oryzae* in addition to improved yield traits. Moreover, transgenic plants overexpressing *OsGRF6*, *OsGRF7*, *OsGRF8*, and *OsGRF9* exhibited enhanced resistance to *M. oryzae*, but showed different alteration of growth. While overexpression of *OsGRF7* led to defects in growth, overexpression of *OsGRF6*, *OsGRF8*, and *OsGRF9* resulted in better or no significant change of yield traits. Collectively, our results indicate that miR396 negatively regulates rice blast disease- resistance via suppressing multiple *OsGRF*s, which in turn differentially control growth and yield. Therefore, miR396-*OsGRFs* could be a potential module to demolish fitness cost in rice blast disease-resistance breeding.

## Introduction

Plants are often targeted by pathogens such as bacteria, fungi, and oomycetes, and as a part of evolution, they developed complex network comprising pathogen-associated molecular pattern- (PAMP)-triggered immunity (PTI) and Effector-triggered immunity (ETI), to counteract the pathogens (Ramirez-Prado et al., 2018). Accumulating evidence indicates that plant microRNAs play important roles in both PTI and ETI responses against pathogens (Seo et al., 2013; Deng et al., 2018). For example, MiR393 regulates host immunity against *Pseudomonas syringae* DC3000 by suppressing auxin signaling through targeting the auxin receptors such as *TIR1*, *AFB2*, and *AFB3* (Navarro et al., 2016). Following studies identified miR160 as a positive regulator, whereas, miR825, miR398b, and miR773 as negative regulators of immunity against bacterial infection in Arabidopsis (Fahlgren et al., 2007; Jagadeeswaran et al., 2009; Li et al., 2010). In tomato, miR482 regulates host defense by targeting coiled-coil-nucleotide-binding leucine-rich-repeat (CC-*NB-LRR*)-encoding transcripts and triggers the production of secondary siRNAs via *RDR6*, which in turn targets other mRNAs of defense related genes (Shivaprasad et al., 2012). In tobacco, nta-miR6019 and nta-miR6020 targets the immune receptor gene *N* that confers resistance to tobacco mosaic virus (Li et al., 2012). In apple, Md-miRLn11 regulates *NBS-LRR* gene expression upon apple leaf spot fungal infection (Ma et al., 2014).

Rice is the most widely cultivated food crop in the world and its production is immensely affected by rice blast disease caused by *M. oryzae* (Baldrich and San Segundo, 2016). Similar to Arabidopsis, rice also involves two layered immune system (PTI and ETI) against *M. oryzae* (Chen and Ronald, 2011). In rice, PTI is mediated by Pattern Recognition Receptors (PRRs) such as CEBiP, LYP4, and LYP6 by effectively chitin recognition, and the second layer of immunity is mostly modulated by nucleotide binding site/ Leucine rich repeat (*NBS-LRR*) proteins (Chen and Ronald, 2011). So far, 102 rice blast resistance (R) genes have been identified, out of which 28 genes were functionally characterized (Li W.T. et al., 2017). Increasing evidence shows that miRNAs modulate responses to *M. oryzae* infection by effectively regulating their target genes (Campo et al., 2013; Li et al., 2014; Baldrich and San Segundo, 2016). For example, Osa-miR7695 is the first miRNA that was identified to promote resistance to *M. oryzae* by negatively regulating its target transcript, *OsNramp6* (Campo et al., 2013). In addition, many new miRNAs, conserved and non-conserved miRNAs were identified to be differentially responsive to *M. oryzae* or its elicitor (Li et al., 2014; Baldrich et al., 2015). Deeper investigation focuses on functionally characterization of each miRNA and its roles in rice blast-disease resistance are disclosed one after another. In the past few years, several groups have identified that miR164a, miR169a, miR319b, and miR444b.2 negatively regulate, whereas, miR160a, miR166k-166h, miR398b, and miR7695 positively regulate rice immunity against the rice blast fungus *M. oryzae* (Campo et al., 2013; Li et al., 2014; Li Y. et al., 2017; Xiao et al., 2017; Salvador-Guirao et al., 2018; Wang et al., 2018; Zhang et al., 2018). However, the roles of other blast fungus-responsive miRNAs remain elusive.

In our previous study, we identified a number of miRNAs that were differentially regulated upon *M. oryzae* infection in susceptible and resistant rice accessions (Li et al., 2014). Among them, miR396 family members were differentially expressed. MiR396d/e-5p and miR396e-3p were induced in the susceptible accession LTH at 12 and 24 h post inoculation (hpi), respectively; whereas, found no change in the resistant accession IRBLkm-Ts. However, miR396c-5p was increased in both LTH and IRBLkm-Ts. Therefore, miR396 might be involved in regulation of rice immunity against *M. oryzae*. However, an in-depth study is essential to elucidate the role of miR396 family members against rice blast fungus due to its multiple isoforms targeting the same group of genes encoding *Growth Regulating Factors* (*GRF*s) (Liu H.H. et al., 2014).

*Growth Regulating Factors* are plant-specific transcription factors defined by the presence of highly conserved WRC and QLQ protein domains that are recognized for their roles in stem, root and leaf development, flower and seed formation, and also maintain growth under adverse environmental conditions (Omidbakhshfard et al., 2015). In rice, the *GRF* transcription factor family comprises 12 members whose expression are epigenetically controlled by miR396 to regulate multiple biological processes (Liu H.H. et al., 2014). For example, miR396-*OsGRF* components are involved in floral organogenesis (Liu H.H. et al., 2014), regulation of grain size and yield (Che et al., 2016; Duan et al., 2016; Gao et al., 2016; Li et al., 2016) and plant architecture (Tang et al., 2018). Recently, evidence has also indicated the role of miR396 during pathogen infection. miR396 expression was significantly reduced in wheat line JD8 (susceptible) and JD8-*Pm30* (resistant line) when infected by *Erysiphe graminis f. sp. tritici* (Xin et al., 2010), and in wheat accession Shan 4445 (susceptible) when infected by *Blumeria graminis* (Wu et al., 2015). Overexpression of Sp-miR396a-5p in tobacco leads to enhanced susceptibility to *Phytophthora nicotianae* infection (Chen et al., 2015). More recently, miR396 was found to regulate defense against hemibiotrophic and necrotrophic fungal pathogens in Arabidopsis (Soto-Suarez et al., 2017). However, the underlying mechanism is largely unclear.

In order to elucidate the role of miR396 in rice immunity against the blast fungus, we analyzed the expression of miR396 isoforms and their target genes in LTH (susceptible) and IRBLkm-Ts (resistant) accessions. Further, we examined the blast disease phenotypes in transgenic lines over-expressing four miR396 isoforms and their target mimicry, respectively. Based on target gene expression in transgenic lines, resistant and susceptible accessions, we selected *OsGRF6*, *OsGRF7*, *OsGRF8*, and *OsGRF9* for further studies. Overexpression of these target genes resulted in enhanced resistance to rice blast fungal infection. In addition, miR396 target mimic and *OsGRF6* overexpressing transgenic lines showed improved yield-traits, in contrast to the transgenic lines overexpressing *OsGRF7* showing growth defects. Overall, our data illustrate that miR396-*GRF*s module regulates rice immunity against blast fungus, which can be combined with improvement of yield-traits. Therefore, miR396-*GRF*s module is highly valuable in disease-resistance and high yield-breeding programs.

## Materials and Methods

### Plant Materials and Growth Conditions

The plant materials used in this study include LTH (*M. oryzae* susceptible) and IRBLkm-Ts (*M. oryzae* resistant) lines. The miR396 overexpression and STTM396 (short tandem target mimic of miR396) transgenic lines were obtained from a previous study (Li et al., 2016) (under Nipponbare, a Japonica accession); and miR396 target mimic (MIM396) and *OsGRF* overexpression transgenic lines were also obtained from a previous study (Gao et al., 2016) (under Yuetai-B, an Indica accession). The rice plants were maintained in the field of Sichuan Agricultural University, Wenjiang, Chengdu. *N. benthamiana* plants were maintained at 22°C with 16/8 h of day/night light in a growth chamber and used for agro-infiltration experiments.

### Plasmid Construction and Genetic Transformation

MiR396 over expression plasmid constructs were provided by Li et al. (2016). Artificial target mimicry sequences of miR396d were inserted into the IPS1 to replace the miR399 target site with primers osa-miR396d – MIMIC FP and osa-miR396d – MIMIC RP (Supplementary Table S3) as described previously (Franco-Zorrilla et al., 2007; Li Y. et al., 2017) and cloned into *Bam*HI–*Bgl*II sites of binary vector 35S-pCAMBIA1300, resulting in over-expressing construct p35S:MIM396d. Reporter plasmids were constructed by inserting the artificial sequences of miR396 target gene site with specific primers (Supplementary Table S3) at the beginning of eYFP codon sequences and cloned into the *Kpn*I site of binary vector 35S-pCAMBIA1300. The constructed plasmids were used for Agrobacterium- (GV3101) mediated transient expression assay in *N. benthamiana.*

### Pathogen Growth and Infection

Four *M. oryzae* strains, Guy11, Zhong1, NC10, and eGFP-tagged Zhong8- 10-14 (GZ8) were used in this study. *M. oryzae* strains were cultured in complete medium (10 g/L D-glucose, 2 g/L Peptone, 1 g/L Yeast Extract, 1 g/L Casamino Acids, 0.1% (v/v) Vitamin Solution, 5% (v/v) Nitrate Salts (120 g/L NaNO_3_, 10.4 g/L KCL, 5.2 g/L MgSO_4_, 30.4 g/L KH_2_PO_4_), 0.1% (v/v) Trace Elements (22 g/L ZnSO_4_⋅7H_2_O, 11 g/L H_3_BO_3_, 5 g/L MnCl_2_⋅4H_2_O, 5 g/L FeSO4⋅7H_2_O, 1.7 g/L CoCl_2_⋅6H_2_O, 1.6 g/L CuSO_4_⋅5H_2_O, 1.5 g/L NaMoO_4_⋅2H_2_O, 50 g/L Na_4_EDTA), 15 g/L agar, pH 6.5) at 28°C with 12-h/12-h light/dark cycles for sporulation. After 2 weeks, spores were collected and the inoculum concentration was adjusted to 1 × 10^5^ spores mL^-1^ for assays. Three-leaf-stage seedlings were used for spray inoculation using 1 × 10^5^ spores mL^-1^ and the disease phenotypes on leaf two were recorded after 5 days post inoculation (dpi). For punch inoculation method (Park et al., 2012), 5 μL of spore suspension (1 × 10^5^ spores mL^-1^) was added at two spots of each leaf, kept in a culture dish containing 0.1% 6-Benzylaminopurine (6-BA). Lesion length was measured after 5 dpi. Relative fungal mass was calculated using DNA concentration of *M. oryzae Pot 2* against the rice genomic *ubiquitin* DNA level by qPCR (Park et al., 2012; Li W.T. et al., 2017. The infection process of fungus and examination of H_2_O_2_ were performed according to Xiao et al. (2003) and Kankanala et al. (2007)), respectively. All quantification analyses of H_2_O_2_ area were conducted in Photoshop according to a previous report (Li W.T. et al., 2017).

### RNA Isolation and Quantitative RT-PCR

Total RNA was extracted from collected samples using TRIzol reagent (Invitrogen). RNA quality and quantity were determined by spectrophotometer and using electrophoresis. Reverse transcription to cDNA was done using the SuperScript first-strand synthesis kit (Invitrogen). To analyze the expression of miRNA, stem-loop pulse RT-qPCR (Varkonyi-Gasic et al., 2007) was performed using the designed stem-loop containing RT and forward primer containing the 5′ end of mature miRNA (forward and reverse) designed according to Varkonyi-Gasic et al. (2007). For internal reference, primers specific to U6 snRNA (Turner et al., 2013) was used. The rice *ubiquitin* (*UBQ*) gene was used as an internal reference for normalizing target gene expression.

### Transient Expression Assay in *N. benthamiana*

*Agrobacterium* strain GV3101 containing the individual expression constructs in the binary vector pCAMBIA1300 was incubated at 28°C overnight in LB media containing specific antibiotics at a 250 r/min shaking incubator. The bacteria were collected at 3000 rpm for 5 min and re-suspended in an MMA buffer (10 mM MES, 10 mM MgCl2, and 100 mM AS). The re-suspended culture was infiltrated into the leaves of *N. benthamiana* for transient expression assay. After 36 hpi, the infiltrated leaves were observed for image acquisition using a NikonA1 Confocal Laser Scanning Microscope (Nikon Instruments, Inc., Chengdu, China) as previously described (Huang et al., 2014). Western blotting analyses were performed following a previous protocol (Chen et al., 2008). Samples were also collected for RNA isolation and RT-qPCR as described above.

## Results

### Differential Responses of miR396 Isoforms to *M. oryzae* in Susceptible and Resistant Accession

**FIGURE 1:**
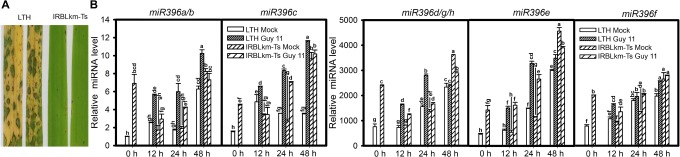
*Magnaporthe oryzae* infection induces differential accumulation of miR396 isoforms in susceptible and resistant rice accessions. **(A)** Representative leaf section of LTH (susceptible) and IRBLkm-Ts (resistant) showing the blast disease phenotype. **(B)** Accumulation of the indicated miR396 isoforms in LTH and IRBLkm-Ts upon *M. oryzae* (or mock inoculated) infection. RNA samples were extracted from leaves collected at different time points and the expression of the indicated miR396 isoforms were performed using Stem-loop RT-qPCR. SnRNA U6 served as the internal reference. Error bars indicate SD from three technical replicates. The letters above the bars indicate significant differences at a *P*-value < 0.01, as determined by a one-way ANOVA followed by *post hoc* Tukey HSD analysis. The experiment was repeated twice with similar results.

MiR396 is a conserved and highly abundant miRNA family reported in most plants including rice and Arabidopsis. The rice genome contains eight loci encoding miR396 (with 5 mature isoforms) distributed on 3 different chromosomes: miR396a, miR396b, miR396c, miR396d, miR396e, miR396f, miR396g, and miR396h (miRBase release 22, Supplementary Figure S1). In order to elucidate the differential responses of miR396 isoforms to *M. oryzae* infection, we analyzed their expression in the susceptible line LTH and the monogenic resistant line IRBLkm-Ts that contains the resistance (*R*) gene *Pi-km* and exhibits high resistance to *M. oryzae* isolates carrying *AVR-Pikm* (Li et al., 2014). Initially, we confirmed the blast disease phenotype of LTH and IRBLkm-Ts by inoculating with Guy11 spores on three-leaf-stage seedlings as described in the Section “Materials and Methods” (Figure 1A). According to the small RNA deep sequencing data from our previous studies, the accumulation of miR396d/e is mostly abundant, followed by miR396c, whereas miR396a and miR396f is very less (Li et al., 2014). In the present study, the accumulation of miR396d, miR396e, and miR396f were increased approximately 3800, 4500, and 3000 folds, respectively, whereas miR396a/b and miR396c expression levels were increased 10 to 12 folds (Figure 1B). Therefore, the data confirmed that the accumulation of different miR396 isoforms was different and are consistent with earlier deep sequencing results. The expression of miR396a/b and miR396c (Figure 1B) were increased at all the three time points tested in LTH, whereas increased only at one or two time points in IRBLkm-Ts. Similarly, miR396d/g/h, miR396e, and miR396f were highly increased at all the three time points in LTH, whereas slightly increased only at 12 and 24 hpi in IRBLkm-Ts. Overall, the data indicate that miR396 isoforms are differentially responsive to *M. oryzae* in rice.

### Overexpression of miR396 Enhances Susceptibility to *M. oryzae*

**FIGURE 2:**
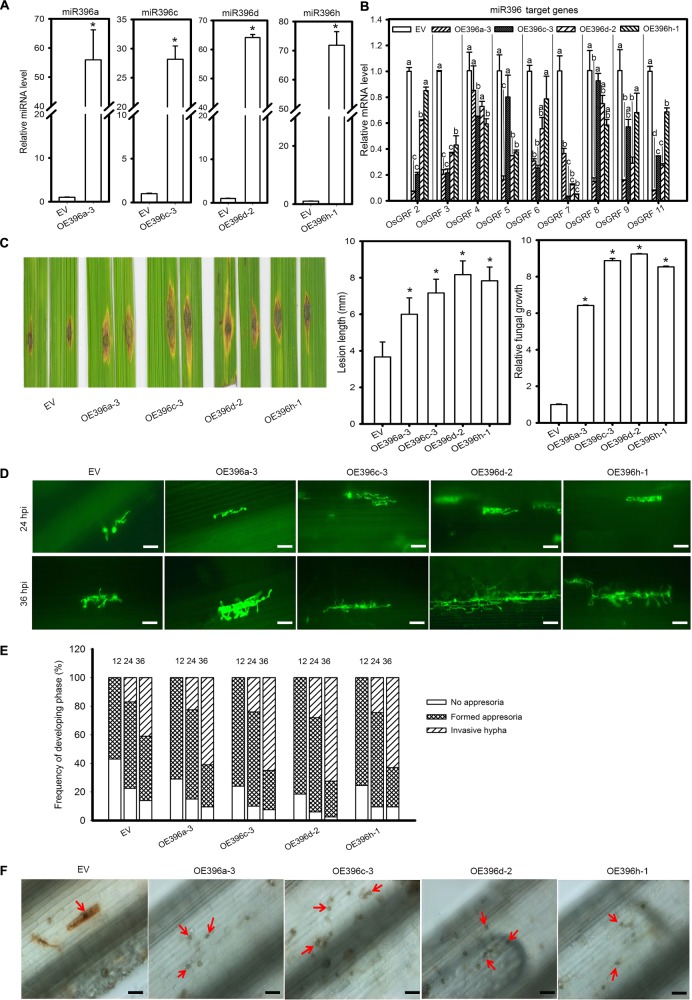
Overexpression of four miR396 isoforms resulted in enhanced susceptibility to *M. oryzae*. **(A)** Accumulation of the indicated four miR396 isoforms in the respective transgenic lines. **(B)** Reduction of *OsGRF* genes in the indicated transgenic lines. **(C)** Blast disease assay on the indicated lines. Punch inoculation of *M. oryzae* strain Zhong1 on 4–5 week old leaves from wild type EV (NPB) and the indicated transgenic lines overexpressing four miR398 isoforms. Disease severity was recorded and evaluated at 5 days post inoculation. Relative fungal biomass is determined by examining the expression level of *M. oryzae Pot2* gene against *OsUbiquitin* DNA level. **(D)** Representative epi-fluorescent images of sheath cells from EV and the indicated transgenic lines infected by eGFP-tagged blast isolate GZ8. Bars = 100 μm. **(E)** Quantification analysis on the progress of fungal infection at 12, 24, and 48 hpi. Around 200 conidia in each line were analyzed. Similar results were obtained in at least two independent experiments. **(F)** DAB staining shows H_2_O_2_ accumulation at the infection sites of the indicated lines at 2 days post inoculation (dpi). Bars = 100 μm. Arrows indicate appressoria. In **(A–C)**, error bars indicate SD (*n* = 3). The letters above the bars indicate significant differences at a *P*-value < 0.01, as determined by a one-way ANOVA followed by *post hoc* Tukey HSD analysis. The asterisk above the bars indicate significant differences between EV and the indicated transgenic lines at a *P*-value < 0.01, as determined by Student’s *t*-test.

Based on the above results we speculated that miR396 might negatively regulate rice immunity against *M. oryzae*. Because all miR396 isoforms target the same group of *OsGRF* genes (Supplementary Figures S1B, S2), we tested four miR396 isoforms in response to the blast fungus. Therefore, we obtained transgenic rice plants overexpressing miR396a, miR396c, miR396d and miR396h (OE396) under Nipponbare (NPB) background. Then, the transgenic lines were subjected to rice blast disease assay. Stem-loop RT-qPCR confirmed the increased accumulation of miR396 isoforms (Figure 2A). Correspondingly, the expression of *OsGRFs* were significantly suppressed in the OE396 lines (Figure 2B). Rice blast disease assay was conducted by punch-inoculation with *M*. *oryzae* strain Zhong1 using detached leaves. Intriguingly, all the OE396 lines exhibited enhanced susceptibility forming lesions larger than control plants (Figure 2C). The lesion lengths were increased up to 1.64, 1.95, 2.22, and 2.13 folds of control plants in OE396a, OE396c, OE396d, and OE396h, respectively (Figure 2C). Fungal biomass quantification also showed that the OE396 lines supported significantly more fungal growth than control plants (Figure 2C).

To understand how OE396 lines supported more fungal growth, we examined the infection process of the eGFP-tagged strain Zhong10-8-14 (GZ8) on sheath cells under epifluorescence microscopy. At 12 hpi, appressoria were formed on sheaths of control and transgenic plants. At 24 hpi, most spores formed invasive hyphae on both control and OE396 lines. At 36 hpi, 65–70% of spores formed invasive hyphae on OE396 lines; by contrast, only ∼50% formed on control lines (Figures 2D,E). These results indicate that overexpression of the four miR396 isoforms highly facilitates the growth of *M. oryzae*.

One typical defense response to pathogen infection is the production of H_2_O_2_ that can be demonstrated by 3, 3′-diaminobenzidine (DAB)-staining. Here, DAB-staining of the *M. oryzae* inoculated leaf sheath showed that the control plants obviously produced H_2_O_2_ in the inoculated sheath at 48 hpi (determined by the staining at the infection site), whereas H_2_O_2_ was hardly detected in the OE396 lines (Figure 2F). The data indicate that overexpression of miR396 may suppress rice defense response, facilitating the growth of *M. oryzae*. Altogether, overexpression of miR396 leads to enhanced susceptibility to *M. oryzae*.

### Transcriptional Regulation of *OsGRF*s by miR396 Isoforms

The potentiality of a miRNA is anticipated on how strong it suppresses target genes. Because overexpression of miR396 leads to suppression of target genes, we speculated to identify the preferential target genes whose expression is more significantly reduced in the OE396 lines. Apart from *OsGRF1*, *OsGRF10*, and *OsGRF12* that we could not detect by RT-qPCR, which might be due to their panicle-specific expression, the expression of the other nine *GRF* genes, including *OsGRF*2, *OsGRF3*, *OsGRF4*, *OsGRF5*, *OsGRF6*, *OsGRF7*, *OsGRF8*, *OsGRF9*, and *OsGRF11*, were significantly suppressed in at least one of the four OE396 lines (Figure 2B). However, the levels of suppression were different among transgenic lines expressing different miR396 isoforms (Figure 2B), indicating preferential targeting of different miR396 isoforms toward different *OsGRF* target sites. While miR396a seems to be the most potent isoform that could significantly suppress all detected *OsGRF*s except *OsGRF4*, miR396c, miR396d and miR396h could significantly suppress six of the nine detected *OsGRF*s (Figure 2B).

**FIGURE 3:**
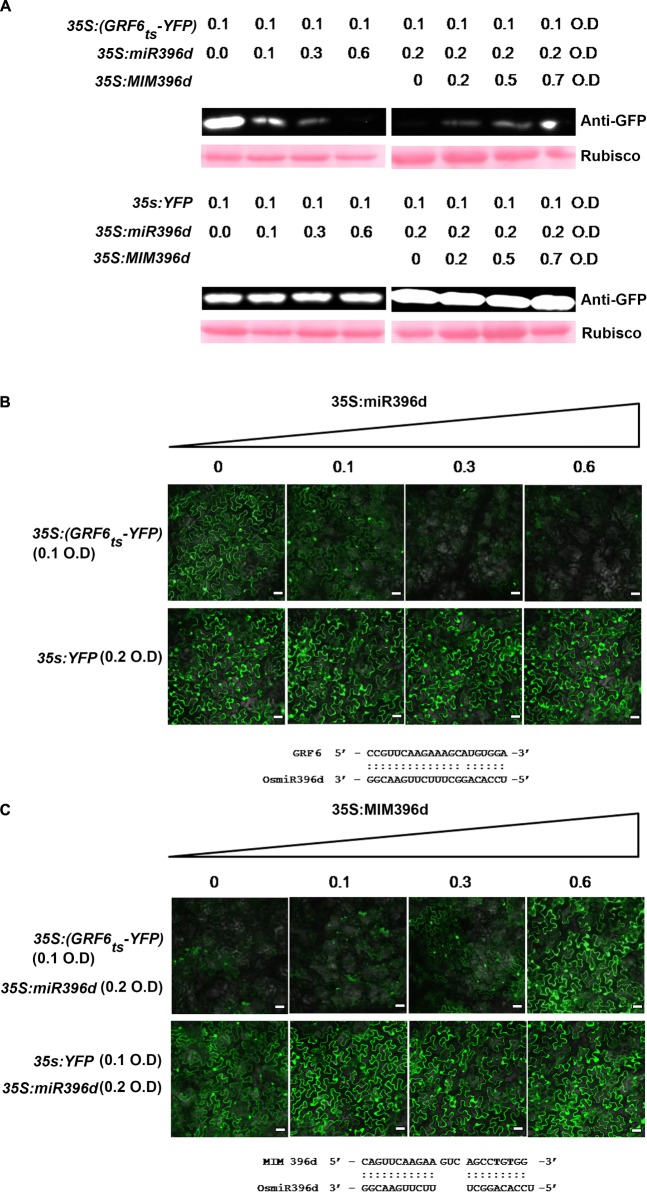
MiR396 represses the expression of its target genes at translational level. **(A)** Western blotting analysis and **(B,C)** confocal images (bars = 25 μm) show that miR396d suppressed the accumulation of GRF6_ts_-YFP but did not affect the protein level of YFP control. The indicated 35S:GRF6_ts_-YFP and YFP-based reporter constructs were transiently expressed alone or co-expressed with miR396d **(B)** or/and the miR396 target mimicry MIM396d **(C)** in *Nicotiana benthamiana* leaves using Agrobacterium-mediated infiltration at the indicated optical density (O.D.) concentration. Protein extracts from the same amount of infiltrated leaves were subjected to Western blot analysis using anti-GFP sera. The Ponceau S stained Rubisco served as loading control **(A)**. The alignments of miR396d with *OsGRF6* target sequence **(B)** and MIM396d with miR396d **(C)** were listed below the images, respectively.

To verify the preferential targeting of *OsGRFs* by different miR396 isoforms, we performed a Yellow Fluorescent Protein (YFP)-based reporter assay, which was transiently expressed in *Nicotiana benthamiana* as described in a previous report (Li Y. et al., 2017). Briefly, we constructed a vector expressing YFP containing the target site of *OsGRF6* at the beginning of eYFP coding sequences (35S:GRF6_ts_-YFP). The Agrobacteria containing 35S:GRF6_ts_-YFP was infiltrated separately or in combination with miR396d in *N. benthamiana* and the protein levels were analyzed by western blotting and by examining the intensity of YFP in the Nikon A1 Confocal Laser Scanning Microscope (Nikon Instruments, Inc., Chengdu, China). When 35S:GRF6_ts_-YFP was expressed alone, it was highly accumulated with intensive signals of YFP (Figures 3A,B). However, when co-expressed with miR396d, the expression was gradually decreased with increased concentration of Agrobacteria harboring miR396d. Conversely, there was no change in the protein concentration or intensity of YFP when the YFP vector without target site or with mutant *OsGRF6* site was expressed at the same conditions (Figures 3A,B). To further validate the targeting of miR396d on 35S:GRF6_ts_-YFP, target mimicry of miR396d (MIM396d) was constructed to trap miR396d in order to reduce its efficiency to bind 35S:GRF6_ts_-YFP. When 35S:GRF6_ts_-YFP was co-expressed along with miR396d and MIM396d, the protein expression was gradually increased with the increased concentration of Agrobacteria harboring MIM396d (Figures 3A,C). The results validated the reporter system.

**FIGURE 4:**
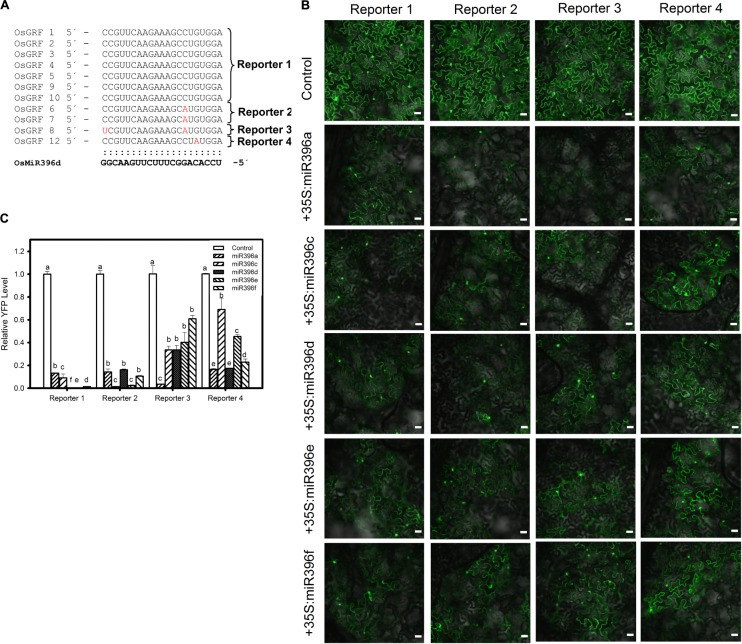
Preferential targeting of miR396 isoforms against *OsGRF*s. **(A)** Multiple alignment of miR396d target sites in *OsGRF*’s. The target genes were classified into four categories (Reporter 1, Reporter 2, Reporter 3, and Reporter 4) based on sequence homology of target site. **(B)** Confocal images show that miR396 isoforms differentially suppresses the protein accumulation of different *OsGRF*’s reporter genes. The indicated YFP-based reporter constructs were transiently expressed alone or co-expressed with miR396 isoforms in *Nicotiana benthamiana.* Bars = 25 μm. **(C)** RNA samples were collected from infiltrated leaves and the transcriptional regulation of *OsGRF*’s homologs by miR396 isoforms were studied using RT-qPCR. Error bars indicate SD from three technical replicates. The letters above the bars indicate significant differences at a *P*-value < 0.01, as determined by a one-way ANOVA followed by *post hoc* Tukey HSD analysis. This experiment was repeated at least three times with similar results.

Next, we conducted the similar experiment with different isoforms of miR396 and *OsGRF*s by analyzing their expression at both translational and transcriptional level. Each of five different plasmids (35S:miR396a, 35S:miR396c, 35S:miR396d, 35S:miR396e, and 35S:miR396f) was co-expressed separately with each of the four reporters (Reporter 1, Reporter 2, Reporter 3, and Reporter 4, Figure 4A) in *N. benthamiana*. Our data showed that all the miR396 isoforms can suppress different target sites of *OsGRFs*, however, we could not distinguish the favorite target site based on the YFP intensity (Figure 4B). Therefore, RT-qPCR analysis was further conducted and showed that Reporter 1 and 2, constituting the most number (nine) of target genes, were highly suppressed when compared to reporter 3 and 4 (each constitutes one gene) (Figures 4A,C), indicating that miR396 family members target some sites stronger than the others. This could be due to the location and number of mismatched nucleotides. Altogether, the above data indicate that different miR396 isoforms differentially regulate different *OsGRFs* at transcriptional level.

### Overexpression of Target Mimicry of miR396 Enhances Resistance to *M. Oryzae* and Improves Yield Traits

**FIGURE 5:**
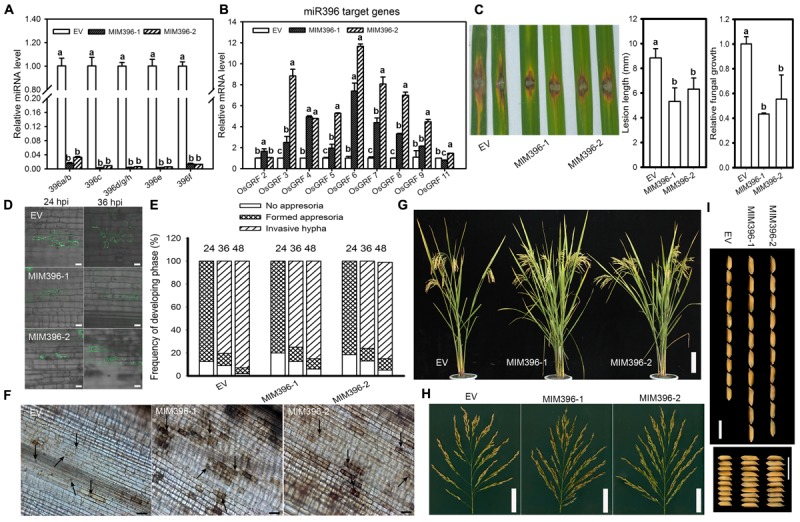
Overexpression of miR396 target mimicry results in enhanced resistance to *M. oryzae* with improved yield traits. RT-qPCR analyses show the expression of miR396 isoforms **(A)** and its target genes **(B)** in control plants (EV, empty vector in YB background) and transgenic lines over-expressing target mimicry of miR396. The accumulation level was normalized to that of the control plants. **(C)** Punch inoculation of 4–5 week old leaves from EV (YB), MIM396-1 and MIM396-2 rice plants show disease severity of *M. oryzae* (Zhong1, 1 × 10^5^ spore/ml conc.) at 5 day post inoculation. Relative fungal biomass is determined by examining the expression level of *M. oryzae Pot2* gene against *OsUbiquitin* DNA level. **(D)** Representative confocal images of sheath cells from EV, MIM396-1 and MIM396-2 infected by eGFP-tagged blast isolate GZ8. Bars = 20 μm. **(E)** Quantification analysis on the progress of fungal infection at 24, 36, and 48 hpi. Around 200 conidia in each line were analyzed. Similar results were obtained in at least two independent experiments. **(F)** DAB staining shows H_2_O_2_ accumulation at the infection sites of the indicated lines at 2 days post inoculation (dpi). Arrows indicate appressoria. Bars = 100 μm. **(G–I)** Comparison of gross plant **(G)**, scale bar = 14 cm; and panicle morphologies **(H)**, scale bar = 5 cm; and seed length and seed width **(I)**, scale bar = 10 mm, of EV (YB) and miR396 mimic (MIM396-1 and MIM396-2) plants. In **(A–C)**, error bars indicate SD from three technical replicates. The letters above the bars indicate significant differences at a *P*-value < 0.01, as determined by a one-way ANOVA followed by *post hoc* Tukey HSD analysis.

To gain more insight on the role of miR396 in rice immunity against blast fungus, we assessed the infectivity of *M. oryzae* against the transgenic plants expressing the target mimicry of miR396 (MIM396) under Yuetai-B (YB) background. The relative expression levels of miR396 isoforms were significantly down-regulated in MIM396 lines (Figure 5A). All the detected target genes, except *OsGRF2* and *OsGRF11*, were significantly increased from 2 to 12 folds in MIM396 lines (Figure 5B). Particularly, *OsGRF*6 level has increased 7 to 12 folds compared to control plants. We next examined the resistance of MIM396 lines against *M. Oryzae* (Zhong1). To our expectation, MIM396 lines showed enhanced resistance as indicated by the lesions smaller than those of the control plants (Figure 5C). The lesion length on the MIM396 lines was significantly reduced when compared to control plants (Figure 5C). Consistently, fungal biomass was significantly decreased in MIM396 lines compared to control plants (Figure 5C). Similar results were obtained when the MIM396 lines were infected with the blast isolate NC10 (Supplementary Figure S3). Correspondingly, we examined the rice blast infectivity in transgenic lines over-expressing STTM396 (Nipponbare background) from a previous study (Li et al., 2016). As expected, the STTM396 lines exhibited enhanced resistance to rice blast disease (Supplementary Figure S4). The lesion length was significantly reduced compared to control plants (Supplementary Figure S4). Quantification of fungal biomass confirmed the lesion length results (Supplementary Figure S4).

We next examined the infection process of GZ8 on sheath cells of control and MIM396 lines. At 12 hpi, appressoria formation was hardly detected; whereas at 24 hpi most spores formed appressoria on control and MIM396 lines. At 48 hpi, ∼95% of spores formed invasive hyphae on control plants; in contrast, only 80–85% formed on MIM396 lines (Figures 5D,E). In addition, DAB-staining showed that the MIM396 lines produced obviously more H_2_O_2_ than the control plants (Figure 5F), which was consistent with the resistance phenotype.

Regarding agronomic traits, the MIM396 lines showed a significant increase in yield-traits compared to control plants, such as the panicle branches, spikelet numbers (Figures 5G,H) and grain size (Figure 5I), which was consistent with a previous report (Gao et al., 2016). To further confirm the growth by field trials, we investigated the agronomic traits in two different experimental locations (Wuhan, ∼N31-E114, and Chengdu, ∼N30-E102, Supplementary Table S2). They showed increased yield traits and were consistent with a previous report (Gao et al., 2016). In total, the above results indicate that blocking miR396 improves both rice blast resistance and yield traits.

### Differential Expression of miR396 Target Genes in Susceptible and Resistant Rice Accession

**FIGURE 6:**
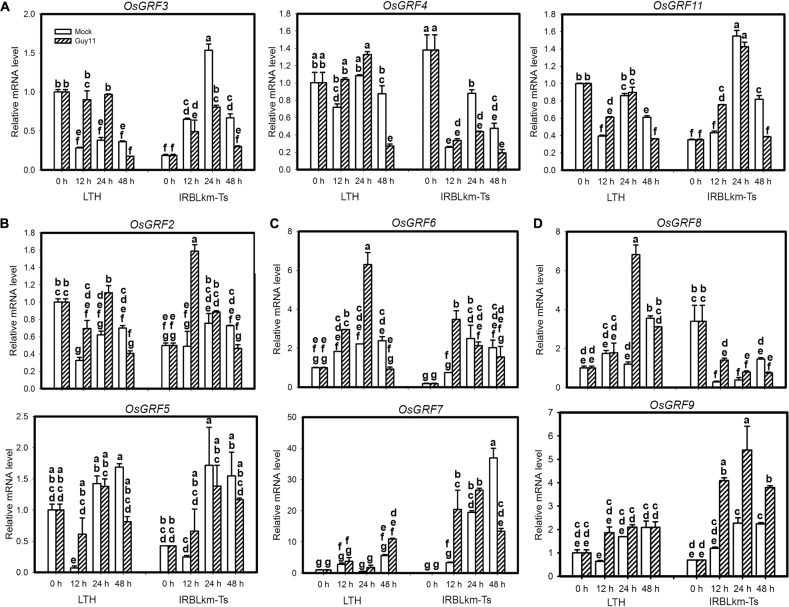
Differential expression of miR396’s target genes in susceptible and resistant accessions upon *M. oryzae* infection. **(A–D)** RT-qPCR analyses show the expression of the indicated *OsGRF* genes in LTH and IRBLkm-Ts at the indicated time points upon *M. oryzae* infection. Relative mRNA level was normalized to that in mock samples before inoculation (0h). Values are means of three technical replicates. Error bars indicate SD. The letters above the bars indicate significant differences (*P* < 0.01) as determined by a one-way ANOVA followed by *post hoc* Tukey HSD analysis.

The above data emphasize the role of miR396 target genes in rice blast resistance. However, it seems that all the 12 *OsGRF*s are regulated by miR396 and it is challenging to identify the one or ones acting in immunity. Therefore, we performed a time course study examining their expression in LTH and IRBLkm-Ts upon *M. oryzae* infection. Based on their expression patterns, they were classified into four groups (Figure 6). The first group included three genes, namely *OsGRF3*, *OsGRF4* and *OsGRF11*, their transcription levels were increased in LTH alone at two time points, whereas decreased at least in one time points in IRBLkm-Ts (Figure 6A). The second group contained *OsGRF2* and *OsGRF5*, their expression pattern in LTH was similar to group 1, whereas, in IRBLkm-Ts the expression levels were significantly increased at 12 hpi, and then decreased or no significant change at later time points (Figure 6B). The third group contained *OsGRF6* and *OsGRF7*, their expression levels were increased in both LTH and IRBLkm-Ts, and however, the expression fold change is much higher compared to first and second group (Figure 6C). The fourth group contained *OsGRF8* and *OsGRF9*, the expression levels were mostly stable or increased at one time point in LTH, whereas in IRBLkm-Ts, the expression levels were significantly increased at least in two time points (Figure 6D). Even though, most genes were up-regulated in LTH, we observed differential expression patterns, indicating that different *GRF*s may act differentially in rice immunity against blast fungus. Genes in group 3 (*OsGRF6* and *OsGRF7*) and group 4 (*OsGRF8* and *OsGRF9*) may positively regulate rice immunity against *M. oryzae*.

### Overexpression of Four *OsGRFs* Enhances Rice Blast Resistance

**FIGURE 7:**
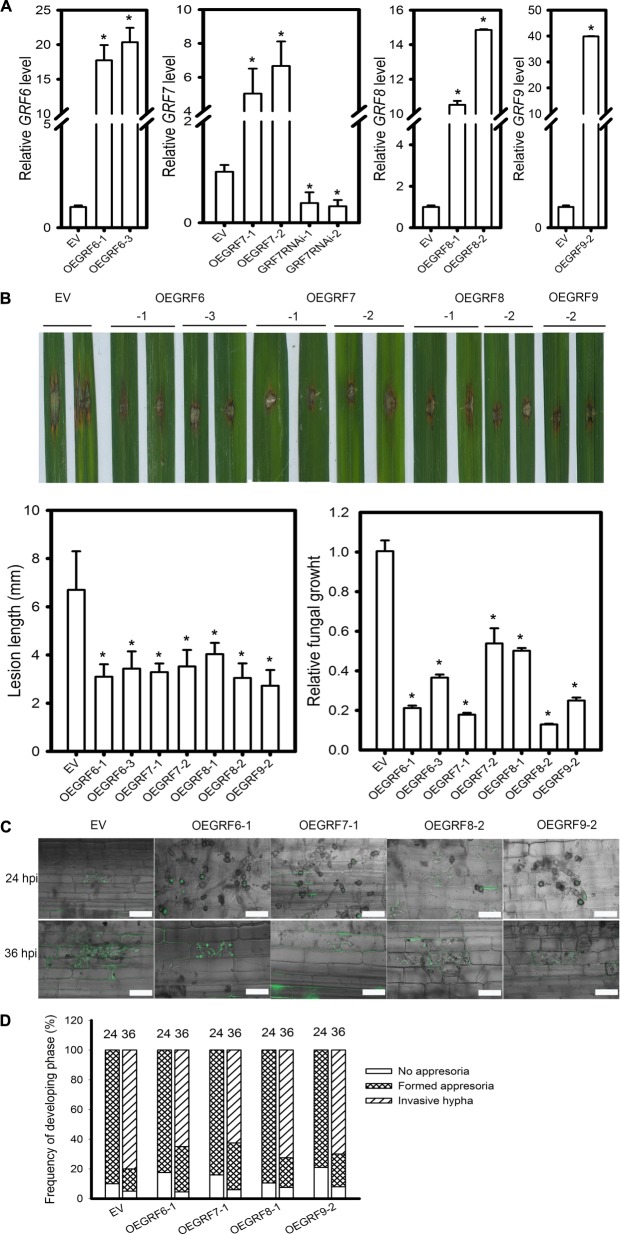
Function of four *OsGRF* genes in rice blast resistance. **(A)** RT-qPCR analyses show the relative expression of *OsGRF6*, *OsGRF7*, *OsGRF8*, and *OsGRF9* in the indicated transgenic lines. *OsUbiquitin* served as the internal reference. The accumulation level was normalized to that of the control plants (EV). **(B)** Disease severity of the indicated lines at 5 days post inoculation of *M. oryzae* strain Zhong1. Punch inoculation was conducted on 4–5 week old leaves of wild type (EV) and the indicated transgenic lines. Relative fungal biomass is determined by examining the expression level of *M. oryzae Pot2* gene against *OsUbiquitin* DNA level. **(C)** Representative confocal images of sheath cells from the indicated lines infected by eGFP-tagged blast isolate GZ8. Bars = 20 μm. **(D)** Quantification analysis on the progress of fungal infection at 24 and 36 hpi. Around 200 conidia in each line were analyzed. Similar results were obtained in at least two independent experiments. In **(A,B)**, error bars indicate SD (*n* = 3). The asterisk above the bars indicate significant differences between EV and the indicated transgenic lines at a *P*-value < 0.01, as determined by Student’s *t*-test.

Based on the target gene expression in LTH and IRBLkm-Ts accession, we speculated that *OsGRF6*, *OsGRF7, OsGRF8*, and *OsGRF9* may positively regulate rice blast resistance. Interestingly, these four genes belong to the same clade in the phylogenetic tree constructed in a previous report (Gao et al., 2016). Therefore, we made transgenic rice lines overexpressing *OsGRF6*, *OsGRF7*, *OsGRF8*, and *OsGRF9* (OEGRF) under Yuetai-B (YB) background and performed blast disease assay. The relative expression levels of *OsGRF6*, *OsGRF7*, *OsGRF8*, and *OsGRF9* in the respective transgenic lines were quantified (Figure 7A). All the OEGRF lines exhibited enhanced resistance to rice blast as demonstrated by the smaller lesions than those in the control plants (Figure 7B). The lesion length on the OEGRF lines was significantly reduced when compared to control plants (Figure 7B). Fungal biomass was also significantly decreased in OEGRF lines compared to control plants (Figure 7B). In addition, we examined the infection process of GZ8 on sheath cells. At 12 hpi, appressoria formation was hardly detected; whereas at 24 hpi most spores formed appressoria on control and OEGRF lines. At 36 hpi, ∼80% of spores formed invasive hyphae on control lines; in contrast, only ∼62.5 to 72.5% formed invasive hyphae on OEGRF lines (Figures 7C,D). Together, all the four OEGRF transgenic lines showed enhanced resistance to blast fungus, among which OEGRF7 was the highest resistant followed by OEGRF6, OEGRF9, and OEGRF8 (Figure 7D). To further confirm the data from OEGRF7, we examined the disease phenotypes of transgenic lines knocking-down *OsGRF7* by RNA interference (GRF7RNAi). The relative *OsGRF7* expression levels were quantified in transgenic lines (Figure 7A). GRF7RNAi lines exhibited enhanced susceptibility as indicated by the larger lesions and more fungal growth that the control plants (Supplementary Figure S5A). In addition, the invasive hyphae of GZ8 formed in GRF7RNAi lines were more aggressive than the control line (Supplementary Figure S5B).

**FIGURE 8:**
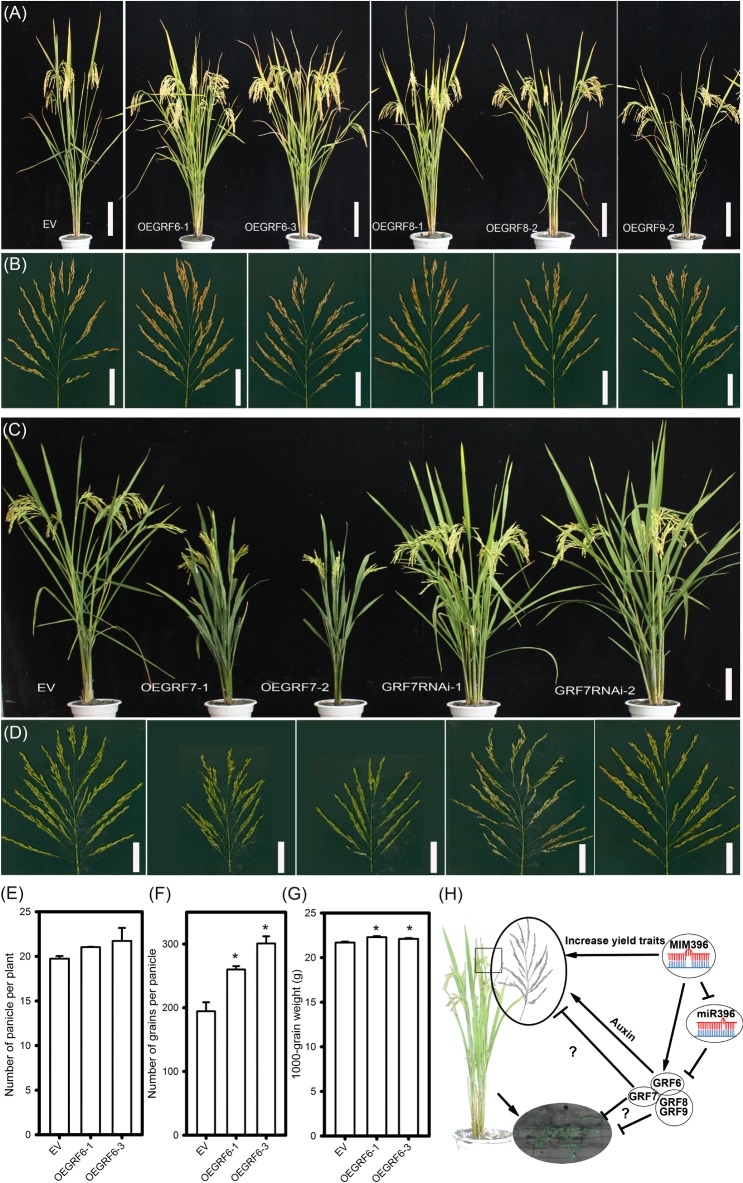
Agronomic traits of transgenic lines overexpressing different *OsGRF* genes and working model for miR396-*OsGRF* in regulation of rice immunity and yield traits. Comparison of gross plant **(A)**, scale bar = 16 cm, and panicle morphologies **(B)**, scale bar = 5 cm, of EV (YB), OEGRF6, OEGRF8 and OEGRF9 transgenic plants. Comparison of gross plant **(C)**, scale bar = 16 cm, and panicle morphologies **(D)**, scale bar = 5 cm, of EV (YB), OEGRF7, and GRF7RNAi transgenic plants. Number of panicles **(E)**, number of grains per panicle **(F)**, and 1000-grain weight **(G)** of *OsGRF6* over-expression lines. The asterisk above the bars indicate significant differences between EV and the indicated transgenic lines at a *P*-value < 0.01, as determined by Student’s t-test. **(H)** Working model for miR396-*OsGRFs* module in balancing yield traits and immunity against the blast fungus.

Regarding agronomic traits, among all the four OEGRF plants, OEGRF6 showed significant increase in yield-traits, such as the number of primary and secondary branches, spikelet numbers (Figures 8A,B and Supplementary Table S2) and larger grain size compared to control plants (Supplementary Table S2), consistent with the previous report (Gao et al., 2016). The number of panicle per plant, number of grains per panicle and 1000-grain weight were also increased (Figures 8E–G). In contrast, OEGRF7 showed reduced height, smaller panicles and less number of secondary branches (Figures 8C,D and Supplementary Table S2). On the contrary, GRF7RNAi lines showed phenotypes comparable to the control plants with higher number of tillers and panicles (Figures 8C,D and Supplementary Table S2). In addition, the growth in field trials was consistent at two sites (Wuhan and Chengdu, Supplementary Table S2). The performance of OEGRF6 was consistent with the earlier reports (Gao et al., 2016), whereas, OEGRF7 exhibited defects in growth. In total, the above results indicate that overexpression of *OsGRF8* and *OsGRF9* enhances rice blast resistance, *OsGRF7* positively regulates rice blast resistance, but negatively regulates growth, and *OsGRF6* positively regulates both rice blast resistance and yield traits.

## Discussion

MiRNAs play important roles in regulating gene expression during plant response to pathogen infection (Seo et al., 2013; Yu et al., 2017). Recently, a large number of *M. oryzae*-responsive miRNAs have been identified through high throughput sequencing (Li et al., 2014; Baldrich and San Segundo, 2016; Zhang et al., 2018). As a result, miRNAs such as miR7695, miR398b, miR160a and miR166k-166h were characterized as positive regulators (Campo et al., 2013; Li et al., 2014; Salvador-Guirao et al., 2018), and miR169a, miR164a, miR319b, and miR444b.2 as negative regulators (Li Y. et al., 2017; Xiao et al., 2017; Wang et al., 2018; Zhang et al., 2018) of rice blast resistance. The miR396 family is highly conserved and contains 8 members in rice (Supplementary Figure S1). Previously, several miR396 family members were found to be responsive to the infection of *M. oryzae* (Campo et al., 2013; Li et al., 2014; Zhang et al., 2015). In the present study, we demonstrated that miR396 negatively regulates rice immunity against the blast fungus *M. oryzae* via suppression of multiple *OsGRF*s. Overexpression of different miR396 isoforms significantly suppressed their target genes *GRF*s (Figure 2B) and led to enhanced susceptibility to *M. oryzae*, which was attributable to reduced defense responses such as less H_2_O_2_ production (Figure 2). In contrast, blocking miR396 via target mimicry significantly up-regulated *OsGRF*s and led to enhanced resistance against *M. oryzae* (Figure 5). The expression of multiple tested *OsGRF* target genes was suppressed at transcriptional level by overexpressing miR396 isoforms (Figures 2B, 3, 4), but upregulated in MIM396 lines (Figure 5B), suggesting that the role of miR396 is through manipulating the expression of *OsGRFs.* Consistently, the transgenic plants overexpressing *OsGRF6*, *OsGRF7*, *OsGRF8*, and *OsGRF9* displayed enhanced resistance against *M. oryzae* (Figure 7). The expression patterns of miR396 also support its negative roles in rice immunity against *M. oryzae*. The accumulation of miR396 isoforms was increased consistently at all the time points in the susceptible accession LTH; however, their expression levels were fluctuated in IRBLkm-Ts (Figure 1). In a previous report (Campo et al., 2013), miR396c expression was decreased, whereas, its target genes *LOC_Os06g10310* (*OsGRF2*) and *LOC_Os03g51970* (*OsGRF6*) were increased upon treatment with elicitors of *M. oryzae*. Similarly, miR396e expression was also down regulated upon infection of *M. oryzae* (Zhang et al., 2015). Thus, the above data demonstrate that miR396 negatively regulates rice blast resistance.

To date, a number of transcription factors, such as WRKY, NAM/ATAF/CUC (NAC), ethylene responsive factor/APETALA2 (ERF/AP2), basic helix-loop helix (bHLH), basic-domain leucine-zipper (bZIP) and MADS box, have been identified to be involved in regulating immunity in crops (Ning et al., 2017). MiR396 targets *GRF*s, which encode transcription factors that involve in plant growth and development (Omidbakhshfard et al., 2015). Accumulating evidence showed that GRFs may play roles in defense responses (Liu J.Y. et al., 2014; Xu et al., 2014; Chen et al., 2015). Here, our data showed that *OsGRFs* were differentially responsive to *M. oryzae* infection in LTH and IRBLKm-Ts (Figure 6). These genes were divided into three clades based on the similarity in amino acid sequences (Gao et al., 2016). Namely *OsGRF1*, *OsGRF2*, *OsGRF3*, *OsGRF4*, and *OsGRF5* belong to clade 1; *OsGRF6*, *OsGRF7*, *OsGRF8*, and *OsGRF9* belong to clade 2; and *OsGRF10*, *OsGRF11*, and *OsGRF12* belong to the clade 3 (Gao et al., 2016). Interestingly, the expression level of genes in the clade 1 (*OsGRF2*, *OsGRF3*, *OsGRF4*, and *OsGRF5*) and clade 3 (*OsGRF11*) were slightly increased (Figures 6A,B), whereas, increased remarkably for the genes in the clade 2 (Figures 6C,D) upon *M. oryzae* infection, indicating that genes of clade 2 (*OsGRF6*, *OsGRF7*, *OsGRF8*, and *OsGRF9*) are highly responsive in comparison to other clades upon *M. oryzae* infection. In addition, *OsGRF6*, *OsGRF7*, *OsGRF8*, and *OsGRF9* might function as positive regulators because their expression levels were significantly up-regulated in the resistant accession IRBLkm-Ts upon *M. oryzae* infection (Figure 6D). Consistently, all the transgenic OEGRF lines from clade 2 exhibited enhanced resistance to *M. oryzae* (Figure 7). Among them, OEGRF7 showed the highest resistance (Figure 7D), which was further confirmed by enhanced susceptibility to *M. oryzae* in GRF7RNAi lines (Supplementary Figure S5). Altogether, the above data describes a novel function of *GRF*s in rice immunity against rice blast fungus.

In rice, different isoforms of miR396 preferentially regulates different *GRF*s involving multiple functions. For example, miR396c decreases salt and alkali stress tolerance by regulating *OsGRF10* and *OsGRF3* (Gao et al., 2010). miR396d affects spikelet development by regulating the expression of *OsGRF6* and *OsGRF10* (Liu H.H. et al., 2014). MiR396b regulates *OsGRF6* for increased grain yield by modulating development of auxiliary branches and spikelets (Gao et al., 2016). Moreover, miR396d (Che et al., 2016), miR396g/h (Duan et al., 2016), miR396c (Hu et al., 2015; Li et al., 2016), regulates *OsGRF4* for controlling grain size and yield, respectively. Here, miR396a, miR396c, miR396d, and miR396h affect yield traits such as seed length, width and 1000-grain weight (Supplementary Table S1) by suppressing six to eight *OsGRF*s (Figure 2B), respectively, consistent with the previous report (Li et al., 2016). Moreover, MIM396 and OEGRF6 lines showed significant improvement in yield traits such as secondary branches, spikelet numbers and grain size (Figures 5, 8 and Supplementary Table S2), confirming the involvement of miR396-*OsGRF6* module in controlling the development of secondary branches in rice inflorescences as suggested in a previous study (Gao et al., 2016). Likewise, miR156 and miR397 have been found to control rice yield. MiR156 regulates rice yield by fine tuning the expression of *SPL* genes (Wang and Zhang, 2017). MiR397 regulates rice yield by increasing spikelet number and grain size by targeting *OsLAC* (Zhang et al., 2013; Zheng and Qu, 2015).

High yield and resistance to pathogens are the major goals in plant breeding. But, they usually act antagonistically and in many cases, the genes that are well known to play defense roles often comes with compromise in plant growth (Ning et al., 2017). For example, mutation in *SPL28* showed enhanced resistance to *M. oryzae*, but led to reduction in yield (Qiao et al., 2010). However, several recent studies have reported that better disease resistance and improved yield could be simultaneously achieved. For example, the paired NBS-LRR receptors, PigmR and PigmS, confer high rice blast disease resistance without yield penalty via an epigenetic regulatory mechanism (Deng et al., 2017). Using pathogen-inducible upstream open reading frame (uORF)-mediated translational control over *AtNPR1*, Xu et al. (2017) obtained engineered rice conferring broad-spectrum disease resistance without fitness costs. Notably, the rice transcription factor Ideal Plant Architecture 1 (IPA1) could improve both grain yield and blast disease resistance through switching DNA binding specificity controlled by phosphorylation (Wang et al., 2018). Furthermore, miRNAs could be ideal candidate to promote both immunity and yield, by fine tuning the expression of their target genes. For example, overexpressing miR444b.1 negatively regulates rice blast resistance with reduced tiller numbers (Xiao et al., 2017), whereas, overexpression of its target gene *OsMADS57* resulted in increased number of tillers (Guo et al., 2013). Similarly, miR164a negatively regulates *M. oryzae* resistance by suppressing *OsNAC60* (Wang et al., 2018), whereas overexpression of another miR164 target, *OsNAC2*, leads to improvement of plant architecture and grain yield in rice (Jiang et al., 2018). In these reports, immunity and yield traits were regulated by different miRNA isoforms or different target genes. Here, we demonstrated a new role for four *OsGRF*s that positively regulated resistance to *M. oryzae*, in addition to their balanced regulation of yield traits (Figures 7, 8). Collectively, we showed that miR396-*OsGRFs* module regulates the trade-offs between plant immunity and yield (Figure 8H). On one hand, miR396 negatively regulates blast disease resistance through suppression of *OsGRF6*, *OsGRF7*, *OsGRF8*, and *OsGRF9*, which in turn, positively regulate blast disease resistance and coordinate development via a yet identify mechanism. Thus, our data provide a potential regulatory module in improvement of blast disease-resistance and yield in rice breeding programs. Since miR396-GRFs module is highly conserved in plants (Liu et al., 2009, 2017; Debernardi et al., 2012; Bazin et al., 2013; Gao et al., 2016), its roles in balancing yield and immunity might be applied in breeding programs for crop improvement.

## Author Contributions

VC, HW, FG, X-LC, Y-PC, G-BL, YZ, X-MY, L-LZ, Z-XZ, J-HZ, Y-GW, and SL performed the experiments. JF, J-QZ, and YL assisted in experiments. VC and W-MW wrote the manuscript. W-MW and S-QL coordinated the overall study and edited the manuscript.

## Conflict of Interest Statement

The authors declare that the research was conducted in the absence of any commercial or financial relationships that could be construed as a potential conflict of interest.

## Supplementary Material

Figure S1 Basic information of miR396 family members. **(A)** Chromosomal location of the eight miR396 family members in rice. The centromere in each chromosome is indicated by a circle. **(B)** Phylogenetic tree derived from the nucleotide sequence comparison. The tree was generated using MEGA 5 by the CLUSTAL W method. The miRBase accession number of the sequences used in the comparison is shown near the miRNA ID. The phylogenetic tree was generated as a consensus of 1,000 bootstrap replicates by the neighbor-joining method. **(C)** Comparison of the mature sequences of miR396 isoforms from miRBase.

Figure S2 Alignment of miR396d with target sequences.

Figure S3 Overexpression of miR396 target mimicry results in enhanced resistance to *M. oryzae* (NC10). Punch inoculation of 4–5 week old leaves from YB (WT), MIM396-1 and MIM396-2 show disease severity of *M. oryzae* (1 × 10^5^ spore/ml conc., NC10) at 5 days post inoculation. Error bars indicate SD from three biological replicates. The letters above the bars indicate significant differences between (EV) and the indicated lines at a *P*-value < 0.01, as determined by a one-way ANOVA followed by *post hoc* Tukey HSD analysis.

Figure S4 Overexpression of STTM396 results in enhanced resistance to *M. oryzae*. Punch inoculation of 4–5 week old leaves from NPB (EV), STTM396-1 and STTM396-2 show disease severity of *M. Oryzae* (1 × 10^5^ spore/ml conc., Zhong1) at 5 days post inoculation. Error bars indicate SD from three biological replicates. The letters above the bars indicate significant differences between EV and the indicated lines at a *P*-value < 0.01, as determined by a one-way ANOVA followed by *post hoc* Tukey HSD analysis.

Figure S5 Silencing of *OsGRF7* resulted in enhanced susceptibility to rice blast fungus. **(A)** Punch inoculation of 4–5 week old leaves from EV (YB) and GRF7RNAi rice plants show disease severity of *M. oryzae* (Zhong1, 1 × 10^5^ spore/ml conc.) at 5 days post inoculation. Relative fungal biomass is determined by examining the expression level of *M. oryzae Pot2* gene against *OsUbiqutin* DNA level. **(B)** Representative confocal images of EV (YB) and GRF7RNAi-1 sheath cells infected by eGFP-tagged blast isolate GZ8, Bars = 20 μm.

Table S1 Agronomic performances of miR396 over expression lines.

Table S2 Agronomic performances of MIM396 and OEGRF transgenic lines.

Table S3 Primers used in this study.
