# Supplementary figures and images for: miR396-OsGRFs Module Balances Growth and Rice Blast Disease-Resistance
Source: Front Plant Sci. 2019 Jan 14;9:1999. doi: 10.3389/fpls.2018.01999 (PMC6339958; doi:10.3389/fpls.2018.01999)

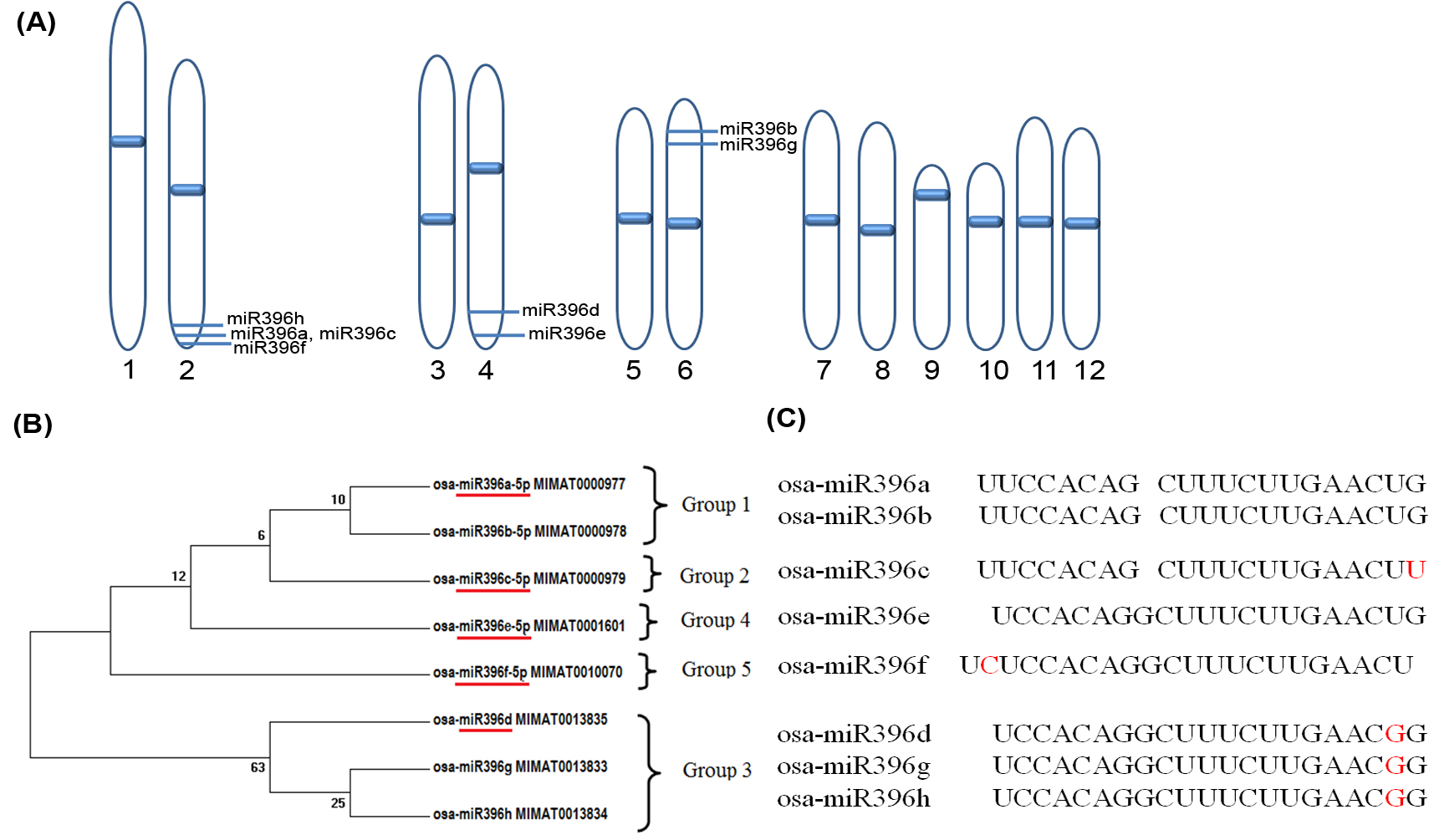

Supplement: Figure S1 — Basic information of miR396 family members. (A) Chromosomal location of the eight miR396 family members in rice. The centromere in each chromosome is indicated by a circle. (B) Phylogenetic tree derived from the nucleotide sequence comparison. The tree was generated using MEGA 5 by the CLUSTAL W method. The miRBase accession number of the sequences used in the comparison is shown near the miRNA ID. The phylogenetic tree was generated as a consensus of 1,000 bootstrap replicates by the neighbor-joining method. (C) Comparison of the mature sequences of miR396 isoforms from miRBase. [file Image_1.JPEG]

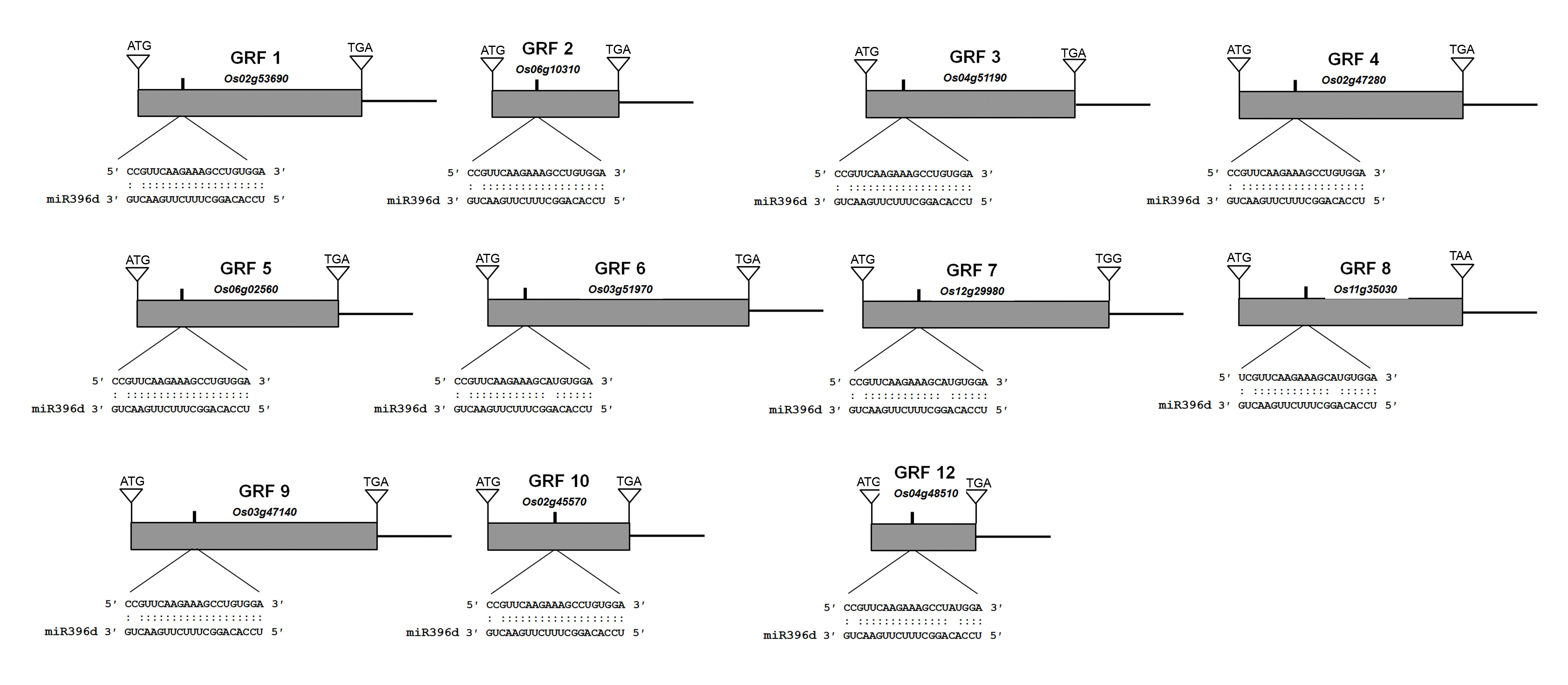

Supplement: Figure S2 — Alignment of miR396d with target sequences. [file Image_2.JPEG]

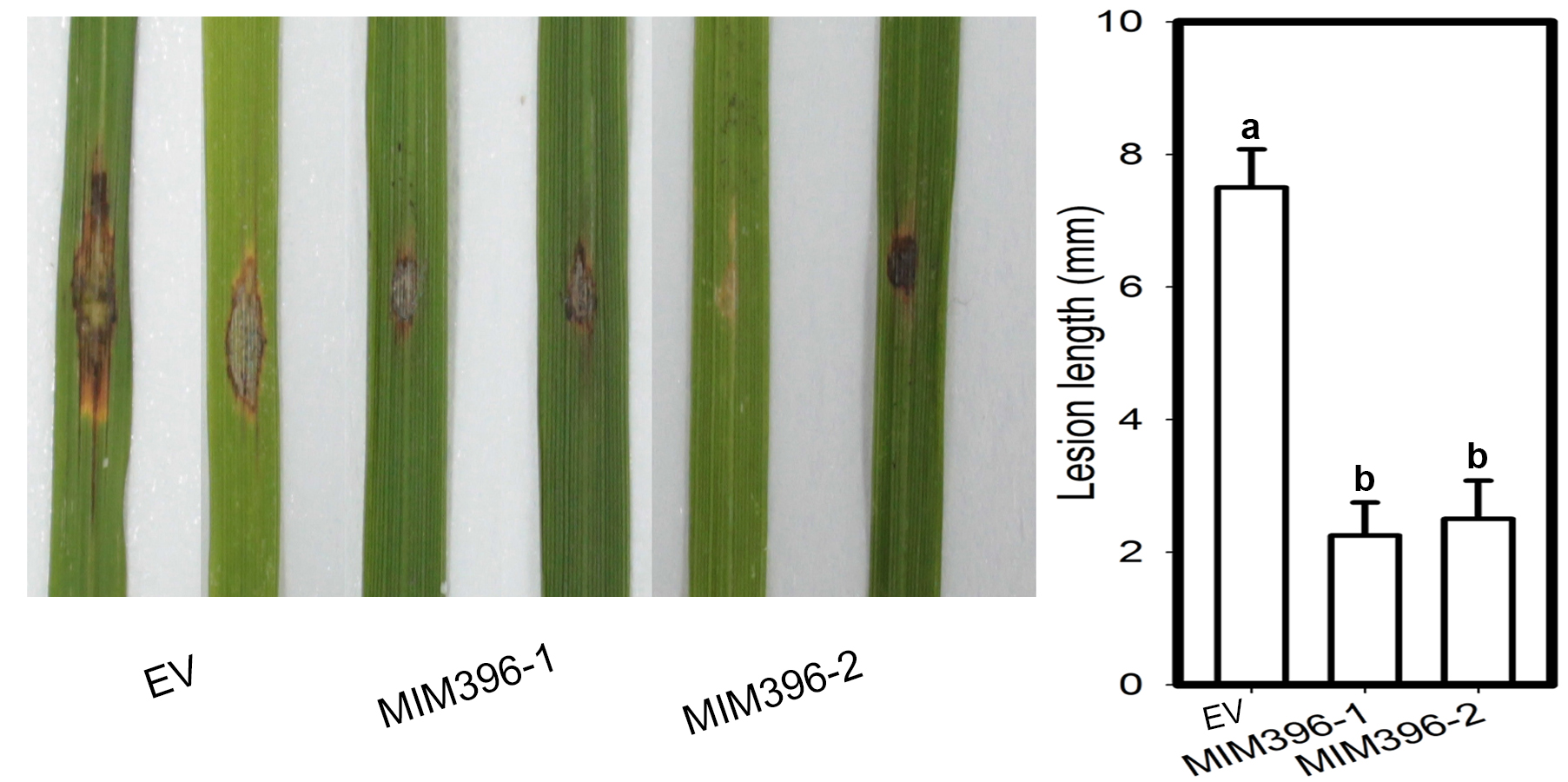

Supplement: Figure S3 — Overexpression of miR396 target mimicry results in enhanced resistance to M. oryzae (NC10). Punch inoculation of 4–5 week old leaves from YB (WT), MIM396-1 and MIM396-2 show disease severity of M. oryzae (1 × 10^5^ spore/ml conc., NC10) at 5 days post inoculation. Error bars indicate SD from three biological replicates. The letters above the bars indicate significant differences between (EV) and the indicated lines at a P-value < 0.01, as determined by a one-way ANOVA followed by post hoc Tukey HSD analysis. [file Image_3.JPEG]

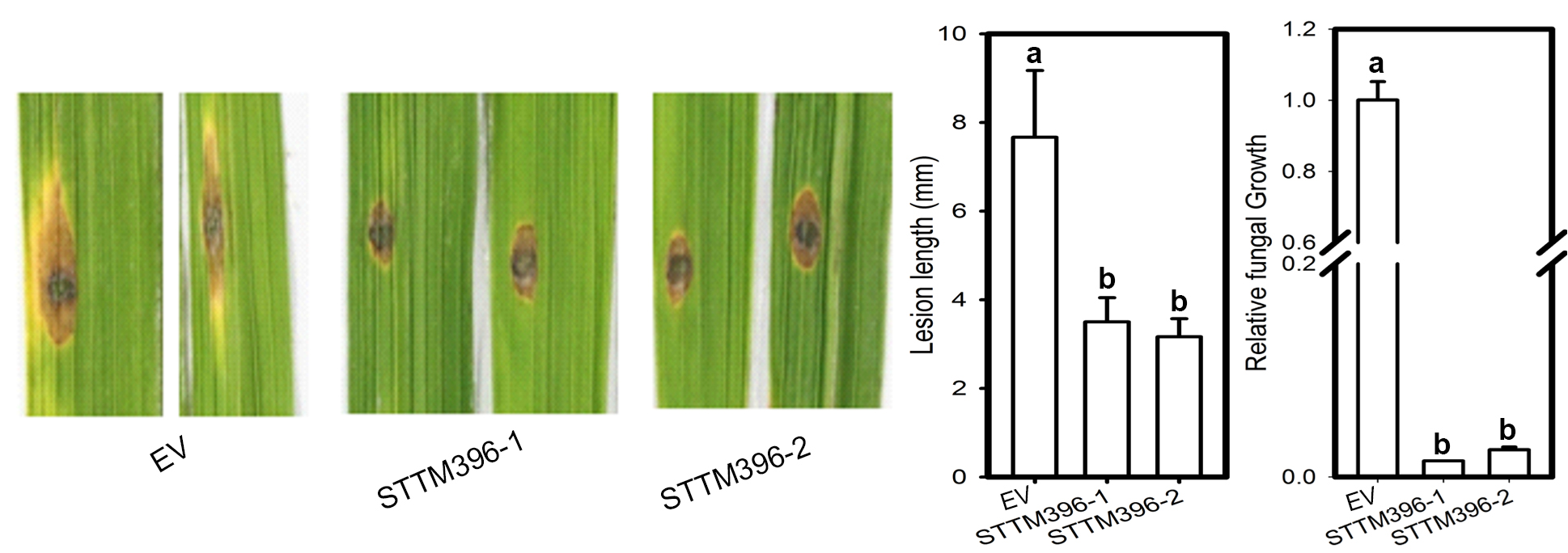

Supplement: Figure S4 — Overexpression of STTM396 results in enhanced resistance to M. oryzae. Punch inoculation of 4–5 week old leaves from NPB (EV), STTM396-1 and STTM396-2 show disease severity of M. Oryzae (1 × 10^5^ spore/ml conc., Zhong1) at 5 days post inoculation. Error bars indicate SD from three biological replicates. The letters above the bars indicate significant differences between EV and the indicated lines at a P-value < 0.01, as determined by a one-way ANOVA followed by post hoc Tukey HSD analysis. [file Image_4.JPEG]

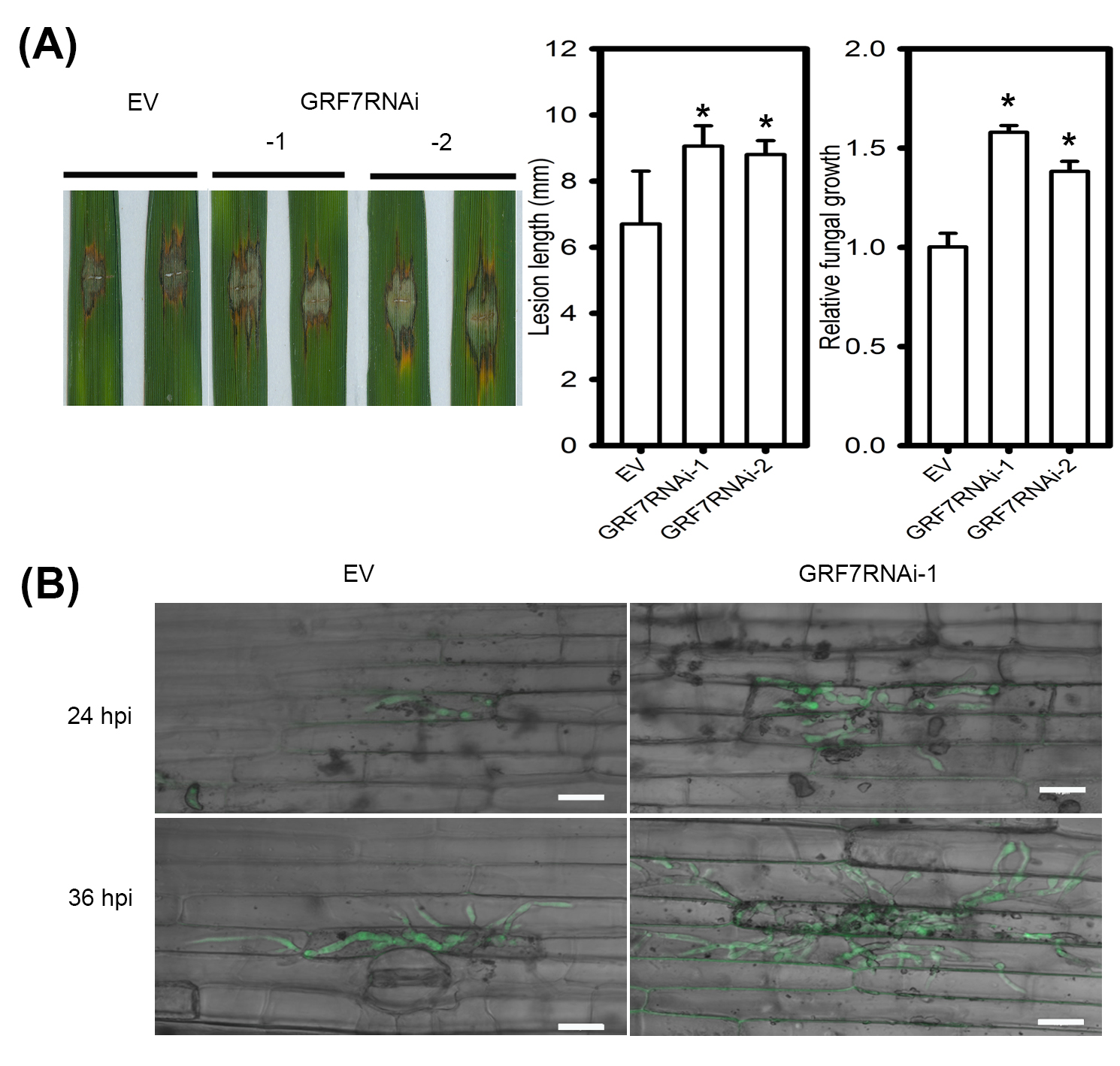

Supplement: Figure S5 — Silencing of OsGRF7 resulted in enhanced susceptibility to rice blast fungus. (A) Punch inoculation of 4–5 week old leaves from EV (YB) and GRF7RNAi rice plants show disease severity of M. oryzae (Zhong1, 1 × 10^5^ spore/ml conc.) at 5 days post inoculation. Relative fungal biomass is determined by examining the expression level of M. oryzae Pot2 gene against OsUbiqutin DNA level. (B) Representative confocal images of EV (YB) and GRF7RNAi-1 sheath cells infected by eGFP-tagged blast isolate GZ8, Bars = 20 μm. [file Image_5.JPEG]
